# Full-Dimensional Ab Initio Potential Energy Surface and Vibrational Energy Levels of Li_2_H

**DOI:** 10.3390/molecules24010026

**Published:** 2018-12-21

**Authors:** Michiko Ahn Furudate, Denis Hagebaum-Reignier, Gwang-Hi Jeung

**Affiliations:** 1 Department of Mechatronics Engineering, Chungnam National University, Daejeon 34134, Korea; furu@cnu.ac.kr; 2 Aix Marseille Univ, CNRS, Centrale Marseille, iSm2, F-13397 Marseille, France; gwang-hi.jeung@univ-amu.fr

**Keywords:** potential energy surface, multi-reference configuration interaction, vibrational configuration interaction, Li_2_H, dilithium hydride, excited states

## Abstract

We built a full-dimensional analytical potential energy surface of the ground electronic state of Li_2_H from ca. 20,000 ab initio multi-reference configuration interaction calculations, including core–valence correlation effects. The surface is flexible enough to accurately describe the three dissociation channels: Li (2s ^2^S) + LiH (^1^Σ^+^), Li_2_ (^1^Σ_g_^+^) + H (1s ^2^S) and 2Li (2s ^2^S) + H (1s ^2^S). Using a local fit of this surface, we calculated pure (*J* = 0) vibrational states of Li_2_H up to the barrier to linearity (ca. 3400 cm^−1^ above the global minimum) using a vibrational self-consistent field/virtual state configuration interaction method. We found 18 vibrational states below this barrier, with a maximum of 6 quanta in the bending mode, which indicates that Li_2_H could be spectroscopically observable. Moreover, we show that some of these vibrational states are highly correlated already ca. 1000 cm^−1^ below the height of the barrier. We hope these calculations can help the assignment of experimental spectra. In addition, the first low-lying excited states of each B_1_, B_2_ and A_2_ symmetry of Li_2_H were characterized.

## 1. Introduction

It is well known that the most characteristic property of the metal atom in a molecular complex is its facility of spontaneously yielding electrons to the surrounding ligands or the surrounding solvent molecules. Among metal atoms, alkali atoms are the most easily electron yielding, as their first ionization potentials are the lowest due to their single-valence electron structure. The electron transfer from the metal atom to the ligand also depends on the ligand’s electron affinity, which in turn depends on the electronic structure of the ligand. The degree of ionicity of a given chemical bonding, as measured by the electron distribution, also depends on the atomic spectra of the metal atom (i.e., its ground and excited states), the electron affinity of the ligand, and the strong perturbation caused by putting the metal atom and the ligand together [1]. The hydrogen atom as ligand makes a moderate ionic bond with metal atoms due to its relatively low electron affinity of about 0.756 eV [2]. Low electron affinity, on the other hand, causes a strong perturbation to many of the excited electronic states in metal hydrides. As a result, the excited states of metal hydride molecules show many interesting properties.

When instead of one metal atom, two metal atoms are brought into the proximity of the ligand, not all valence electrons can participate in the chemical bonding to stabilize the complex. Those valence electrons that do not participate to the bonding constitute the so-called non-bonding electrons, and their role is rather secondary as far as bond energy is concerned. According to the states of the non-bonding electrons, i.e., according to the molecular orbital occupations and the spin states, several molecular states close to the ground state result. The existence of the two metal atoms also makes ionization easy, resulting in a corresponding cation complex. In this work, we studied a small system—Li_2_H, consisting of two lithium atoms and one hydrogen atom—with the most advanced first-principle (ab initio) quantum chemical methods.

Apart from its interesting molecular properties, lithium hydride possesses very useful electrochemical properties, as it is expected to be used as the hydrogen storage material in fuel cells, because lithium is the lightest element that can form solid-state compounds with hydrogen [3].

There are numerous theoretical studies in the literature on the Li_2_H complex, which we briefly summarize here. The first electronic structure calculations for Li_2_H were carried out using the self-consistent field (SCF) and full configuration interaction (CI) techniques by Siegbahn et al. [4] and England et al. [5] in the 1970s. The latter work already contained correlated multi-configurational SCF/CI (MCSCF/CI) calculations on the lowest ^2^A_1_, ^2^B_2_ and ^2^Σ states of Li_2_H. Some of these were confirmed by Talbi et al. [6] in their study of the low-lying states of Li_3_H. In the 1990s, unrestricted Møller-Plesset perturbation methods (UMP) were applied to study the energetics and structure of the electronic ground state of Li_2_H [7] and some low-lying states of Li_2_H [8]. Allouche et al. [9] provided a detailed analysis of the six low-lying states of Li_2_H (two of each A_1_, B_1_ and B_2_ symmetry) using a complete active space self-consistent field approach (CASSCF) and tentatively assigned the origin of two observed bands in an optical absorption spectrum of Li_2_H [10,11]. These last two studies [8,9] and the early study of England et al. [5] are the only reports so far about the electronic states of Li_2_H other than the ground X^2^A_1_ and the first excited states. The first global analytical PES using a bond-order polynomial expansion to best fit 394 ab initio points of full-CI (FCI) quality was due to Maniero et al. in 2010 [12]. More recently, Song et al. [13] developed a potential energy surface (PES) for the electronic ground state of Li_2_H using double many-body expansion fitting based on 3726 ab initio energies calculated at the multi-reference configuration interaction (MRCI) level. Yuan et al. [14] also developed a PES fitting in a many-body expansion manner from 30,000 ab initio energies calculated at the MRCI-F12 level. These PESs have been used in recent time-dependent quantum dynamical studies on the H + Li_2_ (X^1^Σ^+^_g_) reaction [14,15,16,17,18]. It was shown that the rovibrational excitation of Li_2_ inhibits the reaction, and that the Coriolis coupling is highly relevant in this complex-forming reaction via an insertion mechanism.

The experimental data on Li_2_H is scarce. Apart from the two-photon absorption spectrum of Li_2_H [10,11], the only study that has proved the existence of a stable Li_2_H molecule is that of Wu and Ihle [19], from mass spectrometric measurements performed at about 900 K of two gaseous reactions involving Li_2_H. These authors estimated the ionization energy of Li_2_H to be 4.5 ± 0.2 eV and recommended an experimental atomization energy of 89.7 ± 5 kcal∙mol^−1^. However, the latter value was obtained from indirect measurements of equilibrium enthalpies and has to be taken with caution, as we will discuss in the results section. Crooks et al. [20] recorded the photoionization spectrum, showing many vibrational structures, but they could not well resolve them.

Our first aim in this work is to calculate the vibrational energy levels of Li_2_H from a new global analytical PES of the ground electronic state. To achieve this, a grid of high-level ab initio points was fitted using the efficient monomial symmetrization approach (MSA) [21], and the vibrational energies and wave functions were calculated using the MULTIMODE program (version v.3.9, Emory University, Atlanta, GA, USA) [22]. Although the available global analytical PESs are of high quality, none of them includes the core–valence correlation effect. It is, however, expected to be important for alkali species, and is thus desirable in order to approach quantitative accuracy. Indeed, Rosmus and Meyer [23] and Jeung et al. [24] have shown that the inclusion of this effect increases the ionization energy in better agreement with the experimental data, and Jeung et al. [25] have shown that the inclusion of this effect gives better atomic excitation energies too. Therefore, our motivation to build a new global PES was to also include this effect. Last, we also wanted to characterize the first low-lying excited electronic states of Li_2_H and the ground state of Li_2_H^+^.

## 2. Calculation Details

### 2.1. Ab Initio Calculations

We performed ab initio calculations at the Multi-Reference Configuration Interaction Single Double (MRCISD) level using large basis sets for Li and H. These basis sets consist of uncontracted 6s4p2d Gaussian functions for H and 11s6p3d1f (7s6p3d1f) functions for Li. The Li basis set is slightly reduced from that found in [26], where four s-type and four p-type diffuse functions were removed, as this basis set was originally designed to calculate high-lying Rydberg atomic and molecular electronic states. This basis set was optimized (by minimizing the energies) in the atomic CI calculation, including the core–valence and core–core effects, in particular for the Rydberg states. The primitive functions of the H basis set remain unchanged. Unless otherwise mentioned, the present basis set will be referred to in the following as the J99 basis (see Supplementary Materials). The aug-cc-pCVTZ, cc-pCVQZ and cc-pCV5Z basis sets of Dunning [27] (hereafter denoted as ACVTZ, CVQZ and CV5Z, respectively) were also used for comparison with other calculations from the literature. Please note that the (A)CVXZ basis sets are the same as the (A)VXZ basis sets, but augmented with some extra functions to account for core–valence effects. When these effects are neglected in the calculation, the (A)CVXZ basis sets lead to almost identical results as those of the (A)VXZ basis. The Dunning’s basis sets have a larger number of atomic basis functions compared to J99; thus, the former require a lot more computational resources. We compared the computational resources required for different basis sets. Around the equilibrium geometry of Li_2_H, J99 uses about 1/6 as much storage space as CVQZ and 1/50 as much as CV5Z. J99 also uses 1/5 as much total CPU time as CVQZ and 1/26 of the CPU time required by CV5Z. Considering its accuracy (as will be discussed in Section 3.1 and in the Supplementary Materials, Table S2), we adopted J99 to explore the potential energy surfaces in this work.

The reference molecular orbitals (MOs) for the MRCI calculations were obtained by the complete active space (CAS) self-consistent field (SCF) method, with the active space determined by all valence atomic orbitals (FVCAS). The core orbitals of the two lithium atoms were kept inactive in the CASSCF calculations, i.e., they were doubly occupied in all reference configuration state functions (CSFs), but were correlated at the CI level. The core–core and core–valence correlation effects were included by allowing all single and double excitations from all the reference CSFs, leading to about 6 × 10^5^ CSFs. Please note that the Davidson correction was not taken into account in the present study. It usually corrects size-extensivity and size-consistency problems of truncated CI methods (like MRCISD). For the Li_2_H system, we found that this correction was less than 0.2 kcal∙mol^−1^ on the values of the dissociation energies. Davidson correction and other variant types are useful only for cases where the CI is quite truncated, so its use with large-scale CI should be avoided. In addition, Born-Oppenheimer and relativistic corrections, as well as higher-order correlation effects, were neglected. These corrections are expected to be smaller in magnitude compared to the core–core and core–valence correlation effects, and taking these corrections into account is beyond the scope of our study. Since we study the whole surface of the ground state from the global minimum to dissociation channels, as well as some excited states, the choice of multireference methods ensures a proper description of the static correlation.

The valence coordinates $r_{HLi}$ and $\theta_{LiHLi}$ were used to generate a grid of ≈ 2 × 10^4^ MRCI points, which were randomly chosen within the following ranges: $1.5\leq r_{HLi}\leq 12$ [a.u.] and $0<\theta_{LiHLi}<180$ [deg]. All ab initio calculations, as well as Vibrational Configuration Interaction (VCI) calculations (see Section 3.4), were performed using the MOLPRO 2012.1 software (version 2012.1, Stuttgart University, Stuttgart, Germany) [28]. The distribution of points is analyzed and discussed in Section 3.2.

### 2.2. Potential Energy Surface Fitting

We built a global analytical representation of the ab initio PES, based on the monomial symmetrization approach developed by Bowman’s group [21]. This method relies on the use of fitting basis functions that are invariant by permutation of like atoms. It makes it possible to efficiently fit several thousand ab initio energies. The potential is expressed as a sum of symmetrized monomials that are functions of the Morse variables $x_{i}$, which in turn depend on the internuclear distances $r_{ij}$:

(1) $$\left(x_{1},\;x_{2},\;x_{3}\right)=\left(e^{-r_{12}/\lambda },e^{-r_{13}/\lambda },\;e^{-r_{23}/\lambda }\right)$$

where λ is a constant parameter (λ = 3 a_0_), and $(r_{12},\;r_{13},\;r_{23})=\left(r_{Li_{1}Li_{2}},\;r_{Li_{1}H},r_{Li_{2}H}\right)$. The expression for *V* is:

(2) $$V\left(x_{1},\;x_{2},\;x_{3}\right)=\sum_{a+b+c=0}^{k}C_{a,b,c}x_{1}^{a}(x_{2}^{b}x_{3}^{c}+x_{3}^{b}x_{2}^{c})$$

where *a*, *b* and *c* are non-negative integers, and *k* is a positive integer that sets the total order of the polynomial. The fitting procedure relies on a standard linear least-squares algorithm.

### 2.3. Vibrational Energy Calculation

Using the analytical representation of the potential, we computed accurate pure vibrational energy levels (*J* = 0) using the MULTIMODE program [22]. This is based on the following Watson Hamiltonian ${\hat{H}}_{W}$ (in atomic units), which is only valid for non-linear molecules:

(3) $${\hat{H}}_{W}=\frac{1}{2}\sum_{\alpha \beta }{\hat{\pi }}_{\alpha }\;\mu_{\alpha \beta }{\hat{\pi }}_{\beta }-\frac{1}{8}\sum_{\alpha }{\hat{\mu }}_{\alpha \alpha }-\frac{1}{2}\sum_{i}\frac{\partial^{2}}{\partial Q_{i}^{2}}+V\left(Q_{1},...,Q_{N}\right)$$

The first term is the Coriolis coupling term and involves the inverse of the moment of inertia tensor μ, as well as the vibrational angular momentum ${\hat{\pi }}_{\alpha }$, as defined in [29]. The second term is the so-called Watson correction term. The full potential $V\left(Q_{1},...,Q_{N}\right)$ depends on the mass-scaled normal coordinates $Q_{i}$ and is expanded as a hierarchical sum of *n*-mode contributions. For a triatomic molecule (*N* = 3), the potential reads:

(4) $$V\left(Q_{1},Q_{2},Q_{3}\right)=\sum_{i=1}^{3}{V_{i}}^{\left(1\right)}\left(Q_{i}\right)+\sum_{i<j}^{3}{V_{ij}}^{\left(2\right)}\left(Q_{i},Q_{j}\right)+{V_{123}}^{\left(3\right)}\left(Q_{1},Q_{2},Q_{3}\right)$$

where the *n*-mode contributions, $V^{\left(n\right)}$, are given by the full potential with all $Q_{k}$ except $Q_{i}$ equal to zero for *n* = 1, and all $Q_{k}$ except $Q_{i}$ and $Q_{j}$ equal to zero for *n* = 2. The eigenvalues of the Watson Hamiltonian are obtained by a vibrational self-consistent field/virtual state configuration interaction (hereafter named V-CI) approach. For a triatomic molecule, V-CI calculations (*J* = 0) give variational and “exact” vibrational energy levels. As mentioned above, this approach will fail for ground state linear molecules since the Coriolis and Watson terms will diverge, as it may also fail for states lying above the barrier to linearity. However, as long as there are not too many quanta in the bending mode, those high-lying vibrational energy levels should remain accurate.

## 3. Results and Discussion

### 3.1. Ab Initio Calculations for Atomic and Diatomic Species

The electronic energies of atomic species H, Li, and Li^+^ calculated with four different basis sets (see Section 2.1) are reported in the Supplementary Materials (Table S1). The J99 is in excellent agreement with experimental atomic excitation or ionization energies (within less than 0.3% error) when core–valence correlation effects are included. It is shown that the J99 basis is a reasonable basis that requires low computational cost, as mentioned earlier, and has comparable quality to Dunning’s basis (ACVTZ, CVQZ and CV5Z, with or without core–valence effects). The same trend is observed for the diatomic species LiH and Li_2_ (see Table S2): the equilibrium distances, dissociation energies and vertical ionization energies of both diatomics are in reasonably good agreement with experimental values and the recent aug-cc-pVTZ/MRCI-F12 calculations of Yuan et al. [14]. The agreement is better when the core–valence correlation is taken into account, except for the diatomic dissociation energies, for which the differences are less than 1 kcal∙mol^−1^ (= 350 cm^−1^). In the light of these results, one can be confident on the quality of the chosen basis set and method for the calculation on the Li_2_H triatomic complex. Although spectroscopic accuracy is not reached for diatomic dissociation energies, one may expect cancellation of errors for the estimation of reaction enthalpies.

### 3.2. Ab Initio Calculations for the Li_2_H Triatomic Complex

In Table 1, we report our calculated values of equilibrium geometries, atomization, dissociation and ionization energies of the dilithium hydride complex, as well as those from the literature for comparison. As for the atomic and diatomic species, we examine here the effects of the basis sets and core–valence correlation on these properties. Regarding the equilibrium geometries, core–valence correlation effects tend to shorten the equilibrium H–Li bond lengths by ca. 0.02 a.u., and decrease the equilibrium ∠(Li−H−Li) angle by less than 1 degree, regardless of the basis set. The atomization energy, the dissociation energies in both the Li_2_ (^1^Σ_g_^+^) + H (1s ^2^S) and Li (2s ^2^S) + LiH (^1^Σ^+^) channels (E_diss1_ and E_diss2_ respectively) as well as the ionization energy are larger when the core–valence correlation is taken into account. The largest deviations appear on the ionization energies (ca. 0.03 eV = 0.7 kcal∙mol^−1^) and on the atomization energies (ca. 0.3 kcal∙mol^−1^). In the Dunning basis set series ACVTZ<CVQZ<CV5Z, the atomization energies continuously increases, with a difference between ACVTZ and CV5Z of 0.9 kcal∙mol^−1^, regardless of core–valence correlation effects.

In what follows, we compare the present results with calculations from the literature and with the scarce experimental data. Our best calculated equilibrium H–Li bond lengths (3.233 a.u.) and ∠(Li−H−Li) (93.7 degree) differ from less than 0.02 a.u. and 0.9 degree from the most recent calculations [12,13,14]. The comparison of our MRCI/ACVTZ values with the explicitly correlated values of Yuan et al. shows the important influence of the explicit-correlation: distances are shorter by ca. 0.04 a.u. and atomization (resp. E_diss 1_) values are greater by ca. 5 (resp. 3) kcal∙mol^−1^, when explicit-correlation is taken into account. Our values reproduce the experimental data with a 2% error on the equilibrium H−Li bond length, and less than 1% error for the equilibrium ∠(Li−H−Li). Our calculated value of the adiabatic ionization potential (4.671 eV) is in excellent agreement with the experimental value (4.5 eV) and within the experimental error range of ±0.3 eV. Our best estimation of the atomization energy and dissociation energies in the Li_2_ + H and LiH + Li channels (MRCI level considering core–valence correlation with the J99 basis) are 82.05, 58.39 and 25.25 kcal∙mol^−1^, respectively. These are all smaller by 4.6, 3.0 and 2.2 kcal∙mol^−1^, respectively, than the MRCI-F12 values of Yuan et al. [14], and up to 5.85 kcal∙mol^−1^ for the atomization energy compared with the experimental value and Song’s values [13]. We want to critically comment on these values here. First, the apparent excellent agreement between Song’s atomization energy value and the experimental one seems rather artificial, since their PES was scaled to the experimental value. Second, regarding this particular experimental value, it has to be taken with caution, as it was indirectly derived from reaction enthalpies, whereby two different assumptions for the structure of Li_2_H were considered. The originally suggested value of 89.7 ± 5 kcal∙mol^−1^, later corrected by Kim and Herschbach [30] to 87.9 ± 3 kcal∙mol^−1^, was estimated from an average value of two atomization energies assuming a linear Li_2_H geometry, which is obviously not consistent with all calculations from the literature. Furthermore, these two atomization energies were obtained from linear regression of equilibrium constants of two different reactions and from the knowledge of experimental dissociation energies of Li_2_, LiH and Li_3_ molecules. Another estimate from the same authors using a triangular geometry was 82.1 ± 3 kcal∙mol^−1^ (see Table 1), which appears more consistent with our best calculated value.

We also calculated the equilibrium geometries and energies of the excited B_2_, B_1_, and A_2_ states of Li_2_H. We compare our results with calculations from the literature in Table 2. The present calculations were carried out assuming C_2v_ symmetry, as in past studies. The obtained geometries and energies agree well with the past calculations. Among the states we calculated, we found that the lowest 1^2^B_2_ and the highest 1^2^A_2_ excited states are both linear, and the intermediate 1^2^B_1_ has a bent geometry, with a wider Li–H–Li angle than in the ground state geometry. Our excitation energies are in best agreement with the values of Allouche et al. [9] for the 1^2^B_2_ and 1^2^B_1_, and differ by only 630 cm^−1^ from the UMP2 values of Fang et al. [8] over a 17,428 cm^−1^ transition. The present equilibrium Li–H bond length is longer in these states than those in the past studies, except the value of Talbi et al. [6]. The Li_2_H^+^ cation is found to be linear, and our adiabatic and vertical ionization energies are less than 1000 cm^−1^ smaller than those of Allouche et al. [9].

### 3.3. Global Analytical PES of the Ground ^2^A_1_ State of Li_2_H

#### 3.3.1. MSA Fitting of PES

**Figure 1:**
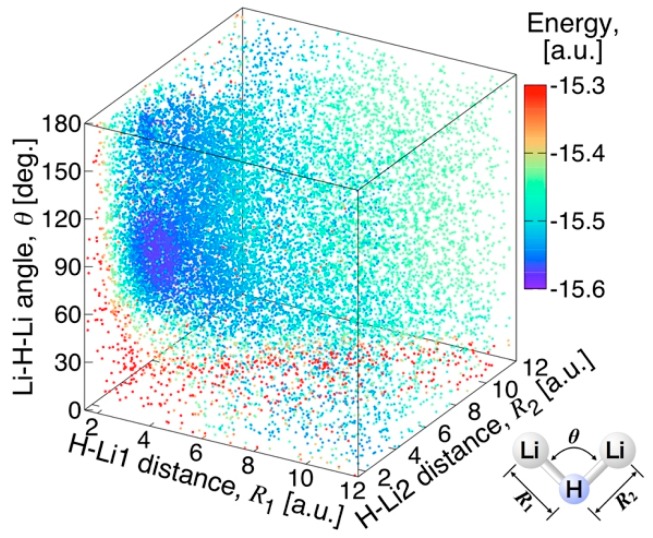
Distribution of the ab initio points for the ground state of Li_2_H.

**Figure 2:**
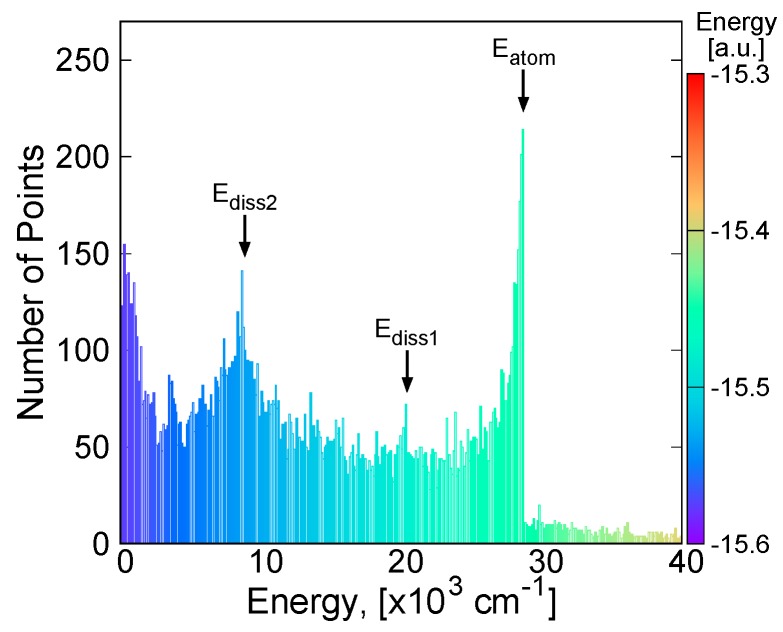
Population histogram of the calculation points over electronic energies. E_atom_, E_diss 1_, and E_diss 2_ correspond to the atomization energy, dissociation energies in the Li_2_H → Li_2_ + H and Li_2_H → Li + LiH, respectively (See text).

The MRCI calculations are carried out for about 20,000 points in the range of 1.5 < *R*_LiH_ < 12 a.u. and 0 < *θ*_LiHLi_ < 180 degrees. The distribution of the calculation points in the parameter space is shown in Figure 1, with the points colored according to the absolute energy value. About half of the points are chosen according to uniform random number generator in the range, and the others are concentrated near the global minimum energy point according to Gaussian distribution. Additionally, several hundred calculation points are added near the minimum energy path (MEP) from the global minimum energy point to the barrier to the linear conformation. Figure 2 shows the statistics on the number of calculation points against the energy measured from the global minimum energy at intervals of 100 cm^−1^. One can see three high peaks in the histogram at the energy intervals of 0~1000, 8500~8600, and 28,400~28,700 cm^−1^. The peak at 0~1000 cm^−1^ indicates that the calculation points are sufficiently concentrated near the global minimum energy value. The energy values of 8600 cm^−1^ (25 kcal/mol) and 28700 cm^−1^ (82 kcal/mol) at the other two peaks correspond to the energies of the LiH + Li asymptote, and the energy of the 2Li + H atomic asymptote, respectively. This implies that these asymptotes are adequately captured in the present ab initio calculations. The H + Li_2_ asymptote is also observed around the energy values of 20,300~20,400 cm^−1^ (58 kcal/mol). The energies higher than the peak at around 28,000 cm^−1^ correspond to geometries in the repulsive region where any of the inter-atomic distances are short.

A fitting function of the potential surface of Li_2_H of the form of Equation (2) was then generated from this grid of MRCI energies using the MSA software (see Ref. [21]). We used *k* = 12 for the highest order of the polynomial of the fitting function, which was found to be optimal, as it leads to a small fitting error for the global PES. The obtained fitting function is a linear combination of 252 monomial basis functions. A Fortran routine for the PES is provided in the Supplementary Materials.

**Figure 3:**
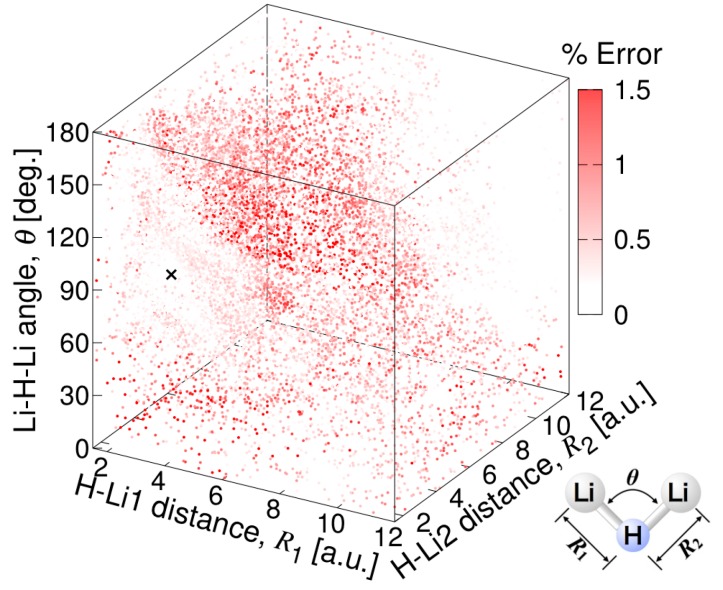
Fitting error distribution of the global analytical PES of Li_2_H in the coordinate space (×: equilibrium geometry).

**Figure 4:**
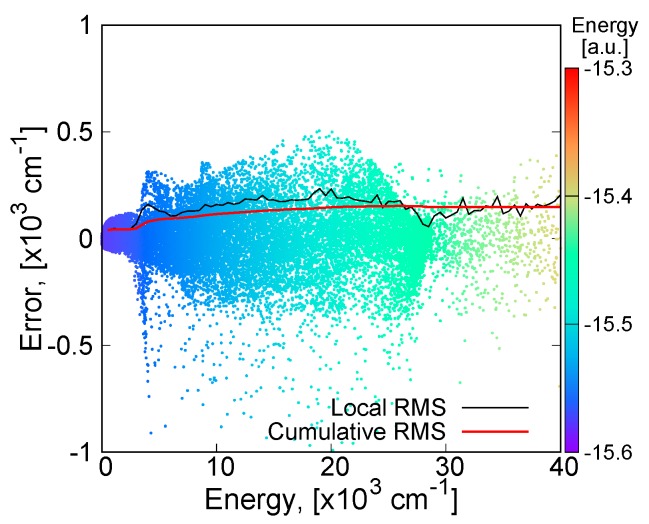
Fitting error distribution of the global analytical PES of Li_2_H in the energy domain (in cm^−1^).

**Figure 5:**
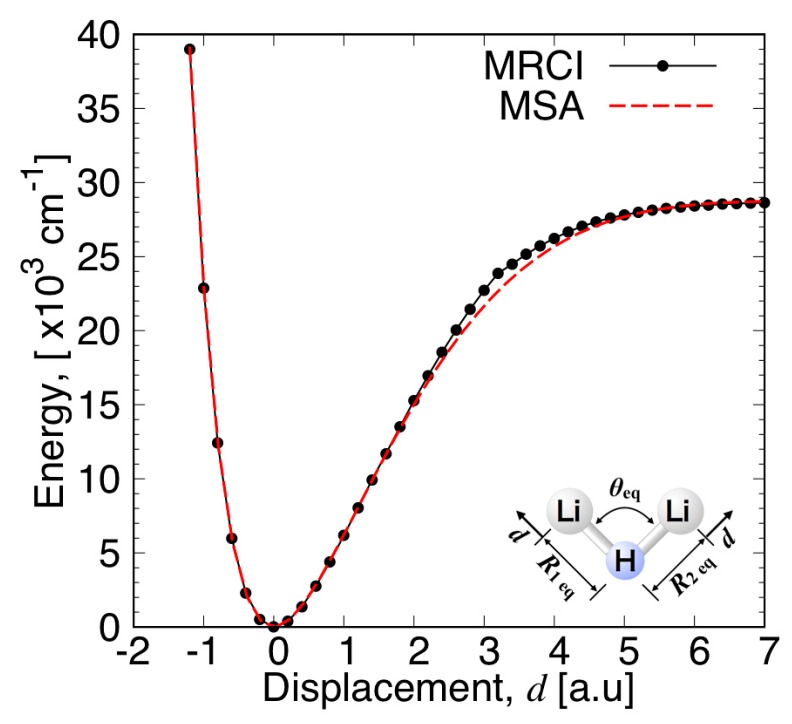
Potential energy curve for the symmetric stretch of Li_2_H at the equilibrium angle.

**Figure 6:**
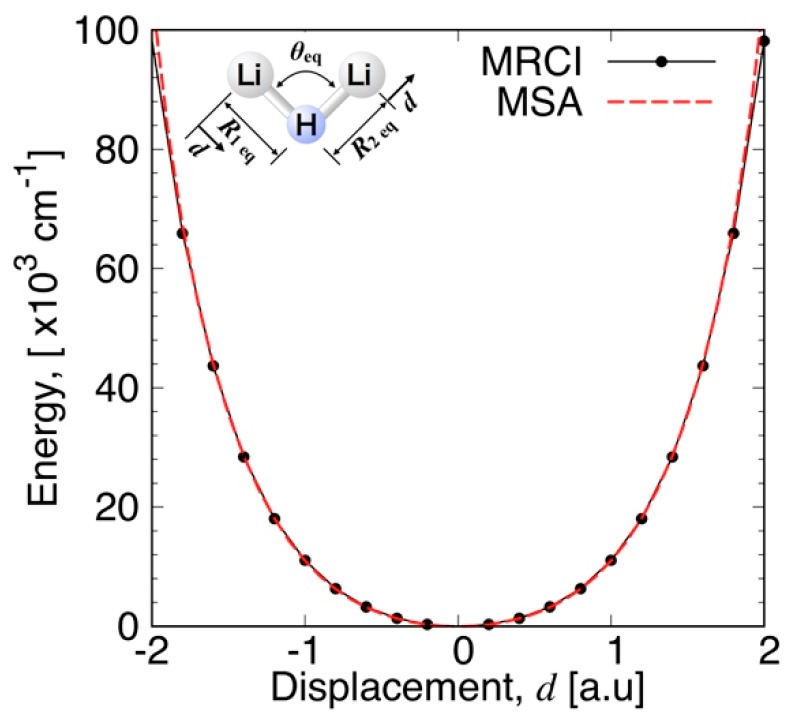
Potential energy curve for the antisymmetric stretch of Li_2_H at the equilibrium angle.

The quality of the present fitting function is examined in Figure 3, Figure 4, Figure 5 and Figure 6. The fitting error distribution in the coordinate space is shown in Figure 3, and in the energy domain in Figure 4. In these figures, the fitting error is defined by the difference between the energy values from the fitting function and from the ab initio calculation. The relative fitting error is obtained from the latter when divided by the ab initio energy values. The root mean square (RMS) of each energy interval, and the cumulative RMS are also shown in Figure 4. The present error is smaller than 0.5% near the equilibrium geometry as seen in Figure 3, and the RMS of the energy intervals below 2500 cm^−1^ is less than 44 cm^−1^. This indicates that the present fitting function represents well the PES around the global minimum. More precisely, the deviation from the MRCI values is about 0.0001 Hartree (22 cm^−1^) at the minimum energy and 0.0017 a.u. and 0.77 degrees for the Li–H bond length and ∠(Li−H−Li) angle, respectively (see Table 1). The rotational constants in GHz determined from the present MSA fitting, (A_e_, B_e_, C_e_) = (22.28, 386.90, 23.64), compare very well with the MRCI values (A_e_, B_e_, C_e_) = (22.06, 392.82, 23.37).

The percentage error of the atomic asymptote is also small, where the two Li–H bond lengths are large and the ∠(Li−H−Li) angle is large, as can be seen in Figure 3. Figure 4 shows that the RMS in the energy intervals in the range from 28,000 cm^−1^ to 29,000 cm^−1^, which is close to the atomization energy limit, is as low as 60 cm^−1^ on average, although the number of calculation points in this energy range is larger. The calculated points with larger errors are concentrated mostly on the geometries having large angles of Li−H−Li and intermediate Li−H bond lengths, as shown in Figure 3. Correspondingly, most of the RMS is cumulated in the energy range from 5000 cm^−1^ to 25,000 cm^−1^, as seen in the plot of cumulated RMS in Figure 4. The RMS averaged for all the calculation points is 154 cm^−1^. To visualize the quality of the MSA fitting over a large energy domain, we plotted a section of the PES corresponding to the symmetric stretch (Figure 5) and to the antisymmetric stretch (Figure 6), where the Li−H−Li angle was fixed at 93.7 degrees. As can be seen from these plots, the analytical function perfectly matches the ab initio values, except in the middle range part of the symmetric stretch potential curve corresponding to energies in the 18,000–27,000 cm^−1^ energy range, where the deviation can locally be as large as 1000 cm^−1^.

#### 3.3.2. Features of the Present Potential Energy Surface

**Figure 7:**
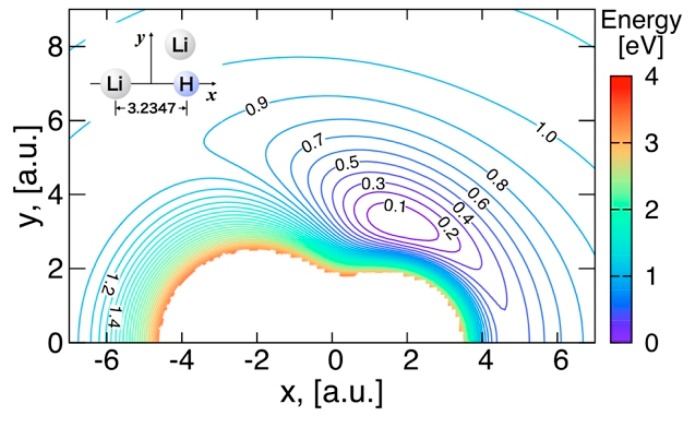
Energy contours when the Li atom moves around a fixed Li−H distance with r(Li−H) = 3.235 a.u. The center of mass of LiH is at the origin. The energy contours are equally spaced by 0.1 eV, starting at the ground state minimum.

**Figure 8:**
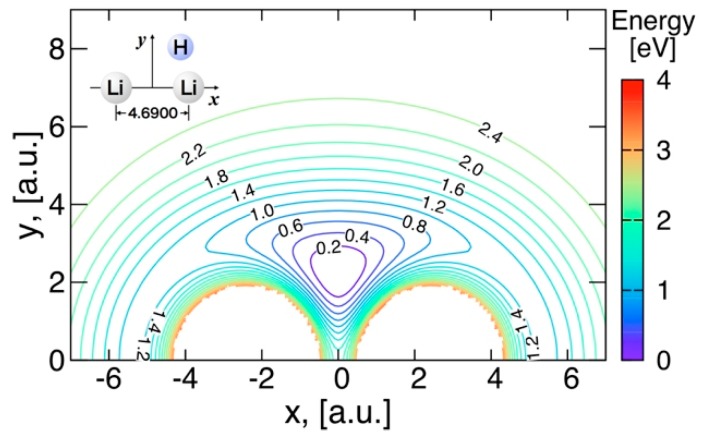
Energy contours when the H atom moves around a fixed Li−Li distance with r(Li−Li) = 4.690 a.u. The center of mass of Li_2_ is at the origin. The energy contours are equally spaced by 0.2 eV, starting at the ground state minimum.

The energy contours in Figure 7 show the potential surface energy when the Li atom is moving around LiH, the distance of which is fixed at the equilibrium distance 3.235 a.u. in the Li_2_H complex. Similarly, the energy contours when the H atom is moving around Li_2_ at the distance of 4.690 a.u. are shown in Figure 8. Minimum energy points appearing in these figures—at (*x*, *y*) = (0.000, 2.228) a.u. in Figure 8, and (*x*, *y*) = (1.783, 3.230) a.u. in Figure 7. Energy contours when the Li atom moves around a fixed Li−H distance with r(Li−H) = 3.235 a.u. The center of mass of LiH is at the origin. The energy contours are equally spaced by 0.1 eV, starting at the ground state minimum.—correspond to the global minimum energy point with the Li–H distance of 3.235 a.u. and the Li−H−Li angle of 92.93 degrees. These contours confirm that the present potential energy surface is fitted smoothly. It can be seen from theses energy contours that the formation of the bent Li_2_H complex from the insertion of H into Li_2_ or from the insertion of Li into LiH proceeds via a barrier-less mechanism, leading to large rate constants for both reactions at low temperature. The leading forces are of different nature in each channel: the major contribution in the attractive long-range potential in the Li + LiH channel is a permanent dipole-quadrupole interaction, whereas it is a permanent quadrupole–quadrupole interaction in the Li_2_ + H channel.

**Figure 9:**
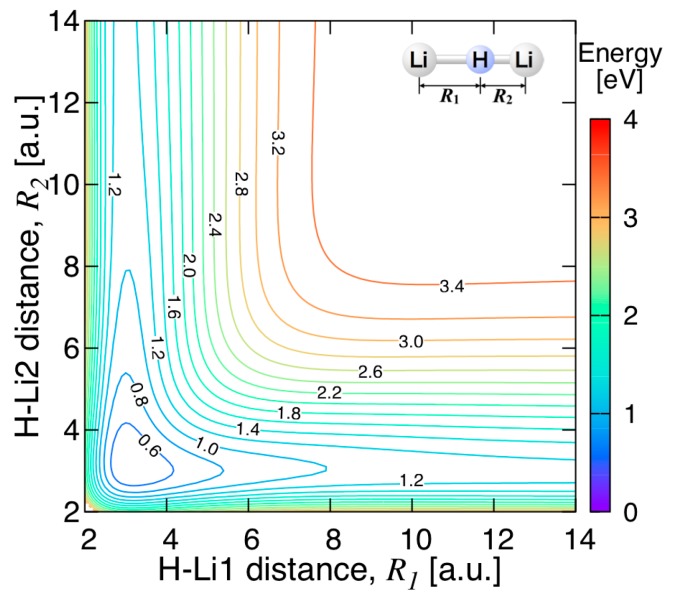
Energy contours for the Li−H−Li linear configuration. The energy contours are equally spaced by 0.2 eV, starting at the ground state minimum.

The energy contours of the present PES for Li−H−Li linear configuration are shown in Figure 9. The minimum energy of 0.4168 eV (3362 cm^−1^) is obtained at the Li–H bond length of 3.137 a.u. This is in good agreement with the 3.172 a.u. value reported by Song et al. [13]. This linear minimum actually corresponds to a saddle point of the full PES, connecting two equivalent bent equilibrium geometries of Li_2_H. The height of the barrier to linearity will be discussed below. The asymptotic energy value of the LiH + Li limit along the MEP is about 1.159 eV (26.7 kcal/mol) for the Li−H bond length of 2.914 a.u., which is calculated as the minimum energy when one of the LiH−Li lengths is fixed at 45 a.u. This value is in reasonable agreement with the dissociation energy of 25.24 kcal/mol for the reaction Li_2_H → LiH + Li obtained from MRCI calculations, as shown in Table 1. Another interesting feature of Figure 9 is that the formation of the linear saddle point is barrier-less, so that the formation of the bent Li_2_H complex can proceed through this saddle point.

**Figure 10:**
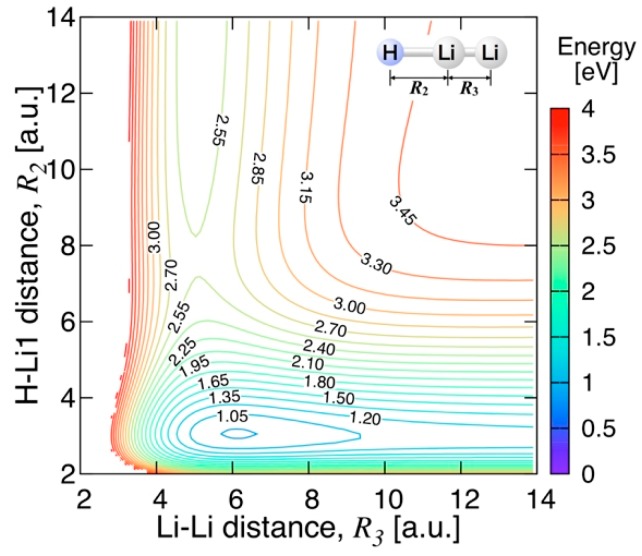
Energy contours for the Li−Li−H linear configuration. The energy contours are equally spaced by 0.15 eV, starting at the ground state minimum.

Figure 10 shows the energy contours of the present PES for the Li−Li−H linear configuration. There exists a local minimum energy point at the Li−H bond lengths of 3.050 a.u. and the Li−Li bond lengths of 6.116 a.u., lying at 0.8902 eV (20.52 kcal/mol) above the global minimum energy. As in Figure 9, this local minimum is a C_∞v_ saddle point of the full PES, connecting two equivalent bent geometries, through an energy barrier of less than 0.9 eV. This agrees well with the results of Song et al. [13], the local minimum energy value of which is 22.15 kcal/mol at the Li−H bond lengths of 3.079 a.u. and the Li−Li bond lengths of 5.960 a.u. The asymptotic energy along the MEP at the Li_2_ + H limit is about 2.449 eV (56.44 kcal/mol), and at the LiH + Li limit about 1.159 eV (26.71 kcal/mol), showing a very good agreement with the MRCI dissociation energies (see Table 1). Contrary to the linear barrier-less Li + LiH reaction, the linear approach of H towards Li_2_ is unfavorable at low collision energies due to a small energy barrier of less than 0.15 eV, as can be seen from the energy contours of Figure 10.

**Figure 11:**
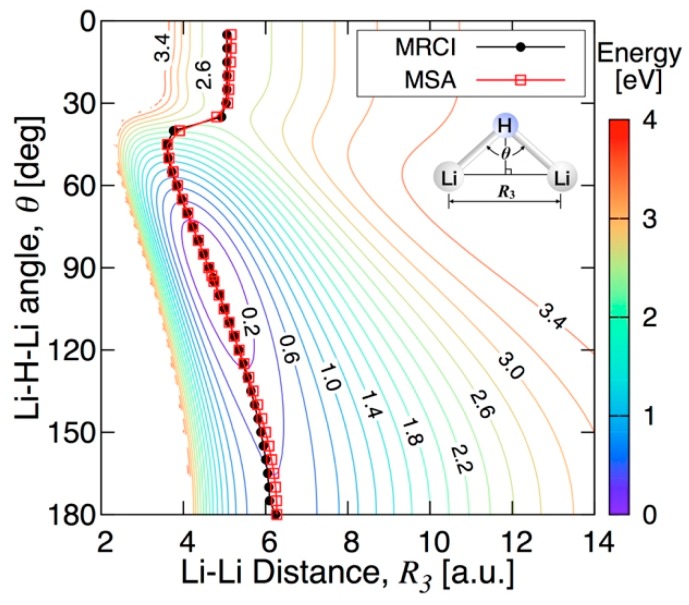
Energy contours of the insertion of H into Li_2_ in C_2v_ symmetry. The energy contours are equally spaced by 0.2 eV, starting at the ground state minimum.

Figure 11 shows the energy contours of the PES for the C_2v_ insertion of H into Li−Li. MEPs from the Li_2_ + H asymptote to the linearity barrier of Li_2_H obtained at the MRCI level and with the fitted PES are also compared in this figure. The agreement between the two paths is very good, with only slight differences for large Li–H–Li angles ( θ > 150 degrees). Please note that the energy values at the low Li−H−Li angles in the MSA fitting results are extrapolated values of the fitting function, because the Li−H distances for these angles are out of the range of the MRCI database used for the fitting. This can be the cause of the underestimation of the energy of the fitted PES at the Li_2_ + H asymptote. The highest value of the energy along the MEP obtained by the MSA fitting is 2.529 eV (20396 cm^−1^ or 58.28 kcal/mol). The energy barrier to linearity of the PES obtained by the MSA fitting is found at the saddle point of Figure 11 at a Li−H bond length of 3.1367 a.u. and a ∠(Li−H−Li) angle of 180.0 degrees with an energy of 0.4168 eV (3362 cm^−1^ or 9.06 kcal/mol). This value is in good agreement with the value of 10.6 kcal/mol found in Ref. [13].

### 3.4. Vibrational Levels

The vibrational levels of Li_2_H are calculated from the present potential energy surface by using the MULTIMODE program [22]. To avoid singularity problems in the MULTIMODE calculations, which occur when the molecular configuration is linear, the vibrational levels are calculated for energy levels lower than the linearity barrier. For a better accuracy of the low-lying vibrational levels, a local MSA fitting for the energies below the linearity barrier was carried out. The local MSA fitting uses 2462 points under the energy of 12,000 cm^−1^, which are taken from the points used for the global PES fitting in the parameter ranges of 40 < θ < 180 degrees for the Li–H–Li angle, 2.7 < r(Li−Li)< 6.0 a.u. for the Li–Li distance, 2.1 < r(Li−H)< 5.72 a.u. for the Li–H distance, and 4.3 < r(Li1−H) + r(Li_2_−H) < 9.8 a.u. for the sum of two Li–H distances. The RMS of the local fitting is 9 cm^−1^, while that of the global fitting for the points below the linearity barrier is 47 cm^−1^. In the MULTIMODE V-CI calculations, the number of basis functions is set to 28 for the bending mode, and 12 for the stretching modes. The Gaussian points for integrations are set to 20. The maximum quantum numbers considered in the one-, two- and three-mode excitations are taken to be 20 for the bending mode and 8 for the stretching modes. With these parameters, 18 vibrational levels (including the ground (0,0,0) level) are converged under the energy of 3362 cm^−1^.

In Table 3 we report the harmonic (ω_i_) and anharmonic (ν_i_) frequencies of the normal modes calculated with the Molpro VCI routine [33] using the grid surface calculated at MRCI and UCCSD(T)-F12 levels on one hand, and from MULTIMODE V-CI with the MSA local and global fits (hereafter MM/MSA) on the other hand. The harmonic frequencies (in cm^−1^) of the bending, symmetric stretching and antisymmetric stretching modes are noted ω_1_, ω_2_ and ω_3_ respectively. 

First, it can be seen that the frequencies at the MRCI level are systematically red-shifted compared to those at UCCSD(T)-F12 level (i.e., they are larger), with a difference lying within a range of 4–10 cm^−1^ for harmonic frequencies, and up to 26 cm^−1^ difference for the anharmonic symmetric stretching. This shows that even two accurate high-level ab initio methods can lead to errors on frequencies that are much greater than the 1 cm^−1^ spectroscopic accuracy. One may expect even larger differences for higher-lying vibrational energy levels. Second, the MM/MSA calculations using the local fit of the PES are in excellent agreement with the MRCI VCI calculations as far as the harmonic frequencies are concerned (less than 3 cm^−1^ error), much better than those with the global PES (up to 23 cm^−1^ error). The same comparison for anharmonic frequencies showed larger errors (up to 35 cm^−1^, up to 4% error), although the comparison is biased since the number of basis functions and maximum quantum numbers in the mode excitations is different in the MULTIMODE and MOLPRO V-CI calculations. In any case, the MM/MSA anharmonic frequencies are lowered by about 5% for the bending and the symmetric stretching mode, and 8% for the antisymmetric stretching mode with both global and local PESs. Similar trends are observed with MOLPRO VCI calculations at UCCSD(T)-F12 level, and a somewhat more pronounced lowering (about 9%) at MRCI level.

**Figure 12:**
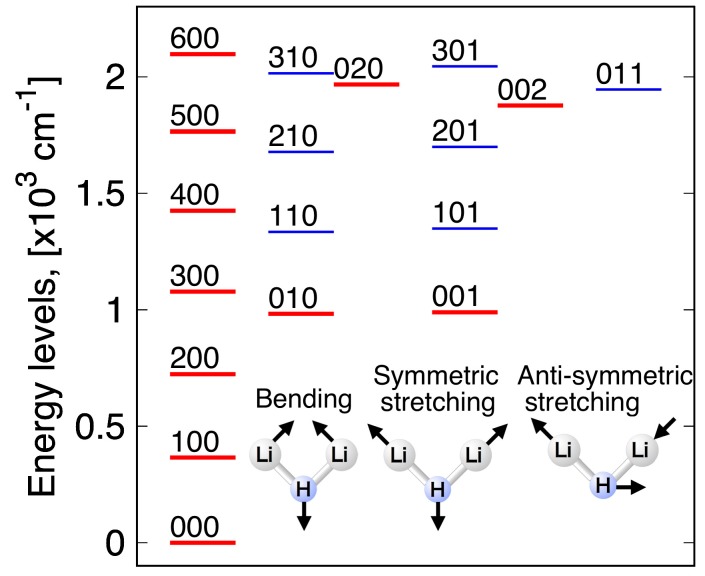
Energy diagram of some low-lying vibrational energy levels (v_1_ v_2_ v_3_) of the ground electronic state of the Li_2_H complex. Levels underlined in red (blue) are one-mode (resp. two-mode) energy levels.

The vibrational quantum numbers and the vibrational energy levels obtained with MULTIMODE are summarized in Table 4. Here, only the vibrational quantum numbers corresponding to the major contribution to the vibrational energy of interest are shown. The calculated zero-point energy is 12,22.8 cm^−1^, and therefore, the linearity barrier measurement from the zero-point energy is 2135 cm^−1^. Under the linearity barrier, 18 vibrational levels are obtained. These energy levels are also schematically drawn in the Figure 12, where one can see that the energy gaps between the one-mode excited levels in red lines are slightly reduced for higher levels. Since the one-mode excited levels of symmetric and antisymmetric modes are similar to each other, their two-mode excited levels in blue with bending modes are also similarly distributed. However, some of the energy gaps between adjacent quantum number levels vary irregularly, due to the coupling of the corresponding vibrational states with other states. We report in Table 5 the first three sets of dominant quantum numbers with their coefficients. As can be seen in this table, the close absolute values of coefficients at the energy of 1876.42 cm^−1^ and 1966.69 cm^−1^ implies that the vibrational states (0,0,2) and (0,2,0) are strongly coupled each other. Although one-mode excited levels of bending mode are not coupled very much with the other excited levels, two-mode excited levels show weak but non-negligible couplings. The bending-symmetric two-mode excited levels are weakly coupled with other two-mode excited levels, and the bending-antisymmetric two-mode excited levels are weakly coupled with three-mode excited levels. These results indicate that even at relatively low vibrational energies below the barrier to linearity, some vibrational states are strongly correlated, and couplings between different modes must be considered in order to get accurate vibrational energy levels.

## 4. Conclusions

A full-dimensional ab initio potential energy surface of the Li_2_H complex was successfully built using the monomial symmetrization approach (MSA) to fit more than 20,000 ab initio MRCI energies, including core–valence correlation effects. These effects mainly impact the geometry (lowering of bond lengths by typically 0.01 Å and angles by 0.3°) and, to a lesser extent, the atomization energy (increasing by 0.3 kcal∙mol^−1^) and ionization potential (increasing by 0.7 kcal∙mol^−1^) of the Li_2_H complex. In some particular cases, the exclusion of the core–valence correlation may appear to give better result in comparison with the experimental data, but this would be purely fortuitous. MRCI data points randomly scattered in a wide range of the valence coordinates enabled us to fit the potential energy surface with good accuracy, not only in the neighborhood of the global minimum energy point, but also for the long-range region approaching to the atomization limit 2Li + H but also LiH + Li and Li_2_ + H dissociation limits. From a local fit of this new potential energy surface, vibrational energy levels were calculated using the MULTIMODE program: 18 levels were found below the barrier to linearity of 3362 cm^−1^, with a maximum of 6 quanta in the bending mode. The contracting coefficients for the vibrational energy levels clearly show that some of the vibrational levels are strongly correlated already well below the barrier, and considering two- and three-mode coupling, appear to be essential to determining accurate vibrational energy levels. We hope our calculations can stimulate the determination of the experimental infrared spectrum of gaseous Li_2_H, which is still lacking in the literature, and help assigning experimental lines. Our future goal is to assign some transition lines attributed to Li_2_H in the photo-ionization spectra measured by Stwalley [20], from superthermal hydrogen atoms colliding with Li atoms and Li_2_ molecules. To achieve this, higher vibrational levels than those analyzed in this work need to be calculated, as well as vibrational levels of the Li_2_H^+^ cation. 

## Figures and Tables

**Table 1:** Comparison of equilibrium geometries and energies of the ground electronic state X^2^A_1_ of Li_2_H.

Li_2_H (X^2^A_1_)	Method	Basis	c.v. ^a^	*R*_*e* Li–H_	∠LiHLi	E_atom_^b^	E_diss 1_^c^	E_diss 2_^d^	E_ion_^e^
				[a.u.]	[degrees]	[kcal∙mol^−1^]	[kcal∙mol^−1^]	[kcal∙mol^−1^]	[eV]
Present	MRCI	J99	Y	3.233	93.70	82.05	58.39	25.25	4.671
	MSA fitting	J99	Y	3.234	92.93	82.11	56.44	26.71	-
	MRCI	CV5Z	Y	3.227	93.60	83.04	58.98	25.34	4.683
	MRCI	CVQZ	Y	3.231	93.66	82.79	58.78	25.26	4.678
	MRCI	ACVTZ	Y	3.240	93.59	82.11	58.24	25.12	4.672
	MRCI	J99	N	3.253	94.04	82.00	58.26	24.94	4.642
	MRCI	CV5Z	N	3.254	93.95	82.70	58.62	25.15	4.646
	MRCI	CVQZ	N	3.256	93.88	82.52	58.48	25.10	4.647
	MRCI	ACVTZ	N	3.262	93.76	81.80	57.99	24.93	4.647
Yuan ^g^	MRCI-F12	AVTZ	N	3.220	94.37	86.64	61.4	27.4	
Song ^h^	Fitting	DMBE/SEC PES	N	3.247	94.31	87.91	65.0	31.3	
	MRCI	V5Z	N	3.252	94.14	84.39			
	MRCI	VQZ	N	3.256	93.95	82.56			
Maniero ^i^	Fitting (Full CI)	pseudo-potential 6-311G (2df, 2pd)	N	3.221	94.65	86.9			
Vezin ^j^	Exp.			3.241	94.70				
Wu ^k^	Exp. (linear)					87.9 ± 3 ^f^			4.5 ± 0.3
	Exp. (bent)					82.1 ± 3			
From Wu ^f^, Verma ^l^ and Stwalley ^m^	Exp.						63.4 ± 3	29.8 ± 3	

^a^ c.v.: core–core and core–valence correlation is included (Y) or not (N); ^b^ Li_2_H → 2Li + H, E_atom_ = 2E(Li) + E(H) − E(Li_2_H); ^c^ Li_2_H → Li_2_ + H, E_diss 1_= E(Li_2_) + E(H) − E(Li_2_H); ^d^ Li_2_H → Li + LiH, E_diss 2_= E(Li) + E(LiH) − E(Li_2_H); ^e^ Li_2_H → Li_2_H^+^, E_ion_ = E(Li_2_H^+^) − E(Li_2_H); Conversion factors: 1 eV = 23.061 kcal∙mol^−1^ = 8065.54 cm^−1^; ^f^ Corrected value in Ref. [30]; ^g^ Ref. [14]; ^h^ Ref. [13]; ^i^ Ref. [12]; ^j^ Ref. [11]; ^k^ Ref. [19]; ^l^ Ref. [31]; ^m^ Ref. [32].

**Table 2:** Equilibrium geometries and excitation energies of the low-lying excited states of Li_2_H and of Li_2_H^+^ with respect to the X^2^A_1_ ground state.

**Li_2_H (1^2^B_2_)**	**Method**	***R*_*e* Li–H_ [a.u.]**	**∠LiHLi [degrees]**	**E–E(X^2^A_1_) [cm^−1^]**	
Present	MRCI	3.174	180.0	3581.7	
Talbi ^a^	MCSCF/SOCI	3.222	180	3382	
Allouche ^b^	CASSCF	3.137	180	3597	
Fang ^c^	UMP2	3.162	179.90	3458.9	
**Li_2_H (1^2^B_1_)**	**Method**	***R*_*e* Li–H_ [a.u.]**	**∠LiHLi [degrees]**	**E–E(X^2^A_1_) [cm^−1^]**	
Present	MRCI	3.035	131.6	8526.6	
Allouche ^b^	CASSCF	2.986	133	8554	
Fang ^c^	UMP2	3.012	145.0	8719.7	
**Li_2_H (1^2^A_2_)**	**Method**	***R*_*e* Li–H_ [a.u.]**	**∠LiHLi [degrees]**	**E–E(X^2^A_1_) [cm^−1^]**	
Present	MRCI	3.167	180.0	17428.3	
Fang ^c^	UMP2	3.140	179.89	16798.6	
**Li_2_H^+^ (1^1^A_1_)**	**Method**	***R*_*e* Li–H_ [a.u.]**	**∠LiHLi [degrees]**	**E–E(X^2^A_1_) [cm^−1^]**	
				adiabatic	vertical
Present	MRCI	3.104	180.0	31654.3	37673.4
Allouche ^b^	CASSCF			32584.8	38392.0
Wu ^d^	Experiment				36295.0

^a^ See Ref. [6]; ^b^ See Ref. [9]; ^c^ See Ref. [8]; ^d^ See Ref. [19].

**Table 3:** Calculated harmonic (ω_i_) and anharmonic (ν_i_) vibrational frequencies of the ground electronic state of the Li_2_H complex using MULTIMODE on the local and global PESs and the VCI routine of MOLPRO at MRCI and UCCSD(T)-F12 levels (ω_1_, ν_1_: bending; ω_2_, ν_2_: symmetric stretching; ω_3_, ν_3_: antisymmetric stretching).

	Vibrational Frequency					
	Harmonic			Anharmonic		
	ω_1_	ω_2_	ω_3_	ν_1_	ν_2_	ν_3_
	[cm^−1^]	[cm^−1^]	[cm^−1^]	[cm^−1^]	[cm^−1^]	[cm^−1^]
MULTIMODE (Global PES) ^a^	395.46	1039.11	1056.73	376.92	983.87	971.79
MULTIMODE (Local PES) ^b^	379.73	1031.62	1079.89	365.00	982.48	989.33
Molpro (VCI, MRCI) ^c^	380.07	1034.22	1079.41	345.29	947.18	978.44
Molpro (VCI, UCCSD(T)-F12) ^d^	376.12	1023.60	1071.50	362.22	973.49	981.20

^a^ At the equilibrium geometry (*R_e_*_LiH_, **∠**LiHLi) = (3.234 a.u., 92.93°); ^b^ (*R_e_*_LiH_, **∠**LiHLi) = (3.234 a.u., 93.84°); ^c^ (*R_e_*_LiH_, **∠**LiHLi) = (3.233 a.u., 93.70°); ^d^ (*R_e_*_LiH_, **∠**LiHLi) = (3.252 a.u., 93.98°).

**Table 4:** MULTIMODE V-CI energies (cm^−1^) of the low-lying vibrational energy levels (below the barrier to linearity) relative to the zero-point energy of nonrotating Li_2_H, using the local PES (see text).

Levels of A_1_ Symmetry					Levels of B_2_ Symmetry				
*i*	v_1_	v_2_	v_3_		*i*	v_1_	v_2_	v_3_	
1	1	0	0	365.00	4	0	0	1	989.33
2	2	0	0	724.46	7	1	0	1	1347.74
3	0	1	0	982.48	10	2	0	1	1699.78
5	3	0	0	1077.97	13	0	1	1	1945.16
6	1	1	0	1333.73	16	3	0	1	2044.91
8	4	0	0	1425.01	Zero point energy: 1222.76 cm^−1^				
9	2	1	0	1678.27					
11	5	0	0	1765.03					
12	0	0	2	1876.42					
14	0	2	0	1966.69					
15	3	1	0	2015.30					
17	6	0	0	2097.23					

**Table 5:** Coupling of the vibrational states of the ground electronic state of the Li_2_H complex. The more strongly coupled states are highlighted in gray.

Energy	First				Second				Third			
[cm^−1^]	v_1_	v_2_	v_3_	Coeff.	v_1_	v_2_	v_3_	Coeff.	v_1_	v_2_	v_3_	Coeff.
365.00	1	0	0	0.9978	1	1	2	−0.0515	2	2	0	0.0197
724.46	2	0	0	0.9961	2	1	2	−0.0513	0	1	0	−0.0335
982.48	0	1	0	−0.9746	0	0	2	0.1509	1	1	0	−0.1277
989.33	0	0	1	0.9697	0	1	1	0.2111	0	1	3	−0.1006
1077.97	3	0	0	0.9926	1	1	0	0.0586	3	1	2	−0.0507
1333.73	1	1	0	0.9520	2	1	0	0.1861	1	0	2	−0.1470
1347.74	1	0	1	0.9699	1	1	1	0.2024	1	1	3	−0.1016
1425.01	4	0	0	0.9860	2	1	0	0.0819	4	1	0	−0.0653
1678.27	2	1	0	−0.9193	3	1	0	0.2344	1	1	0	0.1899
1699.78	2	0	1	0.9697	2	1	1	0.1915	2	1	3	−0.1026
1765.03	5	0	0	−0.9745	3	1	0	−0.1018	5	1	0	0.0881
1876.42	0	0	2	0.6844	0	2	0	−0.5207	0	1	2	0.4024
1945.16	0	1	1	−0.8593	0	2	1	−0.2739	0	0	3	0.2492
1966.69	0	2	0	−0.7742	0	0	2	−0.5558	1	2	0	−0.2176
2015.30	3	1	0	−0.8708	4	1	0	−0.2767	2	1	0	−0.2461
2044.91	3	0	1	0.9682	3	1	1	0.1777	3	1	3	−0.1033
2097.23	6	0	0	0.9548	3	1	0	0.1158	4	1	0	−0.1141

## Supplementary Materials

The supplementary materials are available online.
