# Supplementary material for: Full-Dimensional Ab Initio Potential Energy Surface and Vibrational Energy Levels of Li2H
Source: Molecules. 2018 Dec 21;24(1):26. doi: 10.3390/molecules24010026 (PMC6337435; doi:10.3390/molecules24010026)
Supplement: Supplementary file 1 [file molecules-24-00026-s001.zip › supplementaries_molecules/Molecules_Li2H_Supplementary_Material.pdf]

Supplemental Material

Vibrational Configuration Interaction Calculations For Li_2H From Full-Dimensional *Ab Initio* Potential Energy Surfaces

Michiko Ahn Furudate [1], Denis Hagebaum-Reignier [2,*] and Gwang-Hi Jeung [2]

[1] Department of Mechatronics Engineering, Chungnam National University, Daejeon 34134, South Korea; furu@cnu.ac.kr
[2] Aix Marseille Univ, CNRS, Centrale Marseille, iSm2, Marseille, France; gwang-hi.jeung@univ-amu.fr
* Correspondence: denis.hagebaum-reignier@univ-amu.fr; Tel.: +33-491-288-918

Received: 21 November 2018; Accepted: 18 December 2018; Published: 21 December 2018

Contents

1. J99 basis set 1

2. Calculations on atomic species 2

3. Calculations on diatomic species 3

4. Fortran routines of the MSA PES 6

4.1. Global PES 6

4.2. Local PES 6

References 7

1. J99 basis set and acronyms for Dunning basis sets

The J99 gaussian basis set is given below in the MOLPRO format :

For H:

```
basis={
s,H,19.79, 2.945, 0.7209, 0.2301, 0.08552, 0.019
p,H,2.7,1.0, 0.31, 0.087
d,H,1.1, 0.19
}
```

For Li:

```
basis = {
s,Li,1938.0, 291.5, 66.53, 19.00, 6.305, 2.340, .9599, .4188, .09161, .04140, .01941 ;
    C, 1.4, 0.00054, 0.00417, 0.02114, 0.07898 ;
p,Li,    9.745, 2.187, .6004, .1838, .06625, .02707 ;
d,Li,     .3913, .09734, .03306;
f,Li,    0.0066;
}
```

2. Calculations on atomic species

The electronic energies of atomic species H, Li, and Li^+ calculated by various basis set are summarized in Table S1. For atomic hydrogen, the J99 basis gives the value of -0.499708 [Hartree] for H. Although this value is higher than the values from VQZ and CV5Z, the present basis set is accurate enough to describe the ground-state energy of H and H^-. For the lithium ground state (Li(2s)) and first excited state (Li(2p)), the present basis gives absolute values higher than those obtained with the (A)CVXZ basis basis whether or not the core-valence correlation is included. The absolute energy differences fall in the 0.1-0.3 mH range when core-valence correlation is neglected (3-4 mH when core-valence correlation is included). Ionization energies (resp. Li(2s) <- Li (2p) excitation energies) using the J99 basis are in very good agreement with the Dunning basis sets as they deviate at most of about 60 cm^{-1} (resp. 45 cm^{-1}) with core-valence effects and 2 cm^{-1} (resp. 36 cm^{-1}) without core-valence effects. The core-valence correlation has a clear impact on the accuracy of ionization (resp. Li(2s) <- Li (2p) excitation energies with respect to experimental values since it increases the theoretical values of about 350 cm^{-1} (resp. 150 cm^{-1}). The J99 values are in very good agreement both with the results of Yiannopoulou and Jeung [1] using the full set of J99 and with the experimental values [2] (less than 0.3% deviation on the atomic lithium ionization energy). The difference of the ionization energy value predicted by the present basis and the experimental value is less than 50 cm^{-1}. These results justify the use of the present basis set and the MRCI method considering core-valence correlation.

Table S1. Comparison of energies of atomic H, Li and Li^+ species.

H	Method	Basis[§]	c.v[#]	E [a.u.]		
Present	MRCI (HF)	J99	-	-0.499708		
		V5Z	-	-0.499995		
		VQZ	-	-0.499948		
**Li (2s)**	**Method**	**Basis**	**c.v.**	**E [a.u.]**	**E-$E_{Li(2s)}$[cm^{-1}]**	**E_{Li+}-E[cm^{-1}]**
Present	MRCI	J99	Y	-7.472929	0	43440
		CV5Z	Y	-7.477386	0	43468
		CVQZ	Y	-7.476682	0	43451
		ACVTZ	Y	-7.474562	0	43380
		J99	N	-7.432571	0	43085
		CV5Z	N	-7.432723	0	43085
		CVQZ	N	-7.432695	0	43085
		ACVTZ	N	-7.432682	0	43083
Yiannopoulou[a]	MRCI	J99[c]	Y	-	0	43441
Kelly[b]	Exp.	-	-	-	0	43487
**Li (2p)**	**Method**	**Basis**	**c.v.**	**E [a.u.]**	**E-$E_{Li(2s)}$[cm^{-1}]**	**E_{Li+}-E[cm^{-1}]**
Present	MRCI	J99	Y	-7.404830	14946	28498
		CV5Z	Y	-7.409485	14902	28565
		CVQZ	Y	-7.408750	14909	28542
		ACVTZ	Y	-7.406574	14922	28458
		J99	N	-7.364749	14885	28200
		CV5Z	N	-7.365063	14850	28236
		CVQZ	N	-7.365027	14851	28234
		ACVTZ	N	-7.364990	14857	28226
Yiannopoulou[a]	MRCI	J99[c]	Y	-	14916	28525
Kelly[b]	Exp.	-	-	-	14904	28583
**Li^+ (1s)**	**Method**	**Basis**	**c.v.[%]**	**E [a.u.]**		
Present	MRCI	J99	Y	-7.275002		
		CV5Z	Y	-7.279332		
		CVQZ	Y	-7.278701		
		ACVTZ	Y	-7.276906		
	HF[d]	J99	N	-7.236262		
		CV5Z	N	-7.236411		
		CVQZ	N	-7.236384		
		ACVTZ	N	-7.236380		

[§] VXZ (resp. AVXZ) basis correspond to cc-pVXZ (resp. aug-cc-pVXZ) basis sets of Dunning [3]

[#] c.v.: core-core and core-valence correlation is included (Y) or not (N); [%] For Li^+, only core-core correlation is included (Y). When this is not included, the MRCI value is the HF value.

[a.] Ref. [1] , [b] Ref. [2], [c] The original J99 basis of Ref. [1] consists of 15s10p6d3f->(11s10p6d3f) for Li and 6s4p2d->(3s4p2d) for H.

3. Calculations on diatomic species

The minimum energies of the electronic ground state of Lithium hydride and Lithium dimer are calculated at the MRCI level with the ACVTZ, CVQZ, CV5Z and J99 basis sets, and the results are compared in Table S2. Similarly to the calculations of atomic electronic energies, the absolute energy of the ground state minimum of LiH ($X^1\Sigma$) and Li_2 ($X^1\Sigma_g^+$) predicted with the J99 basis is the highest

among the basis sets whether or not core-valence correlation is included. The equilibrium bond lengths, the dissociation energies, and the vertical ionization energies are also compared with the calculated results in past studies and the experimental data. The equilibrium bond lengths obtained with the CVQZ and CV5Z basis (with no core-valence correlation) agree well with those of the VQZ and V5Z calculations in Song et al. [4], as well as Jasik et al. [5,6] studies. When the core-valence correlation is considered, the calculated equilibrium bond lengths are shorter than those from calculation without core-valence correlation, being closer to the experimental value. The bond lengths calculated with the J99 basis considering core-valence correlation compare well with experimental values (0.06 a.u difference for LiH and 0.001 a.u. for Li_2). The dissociation energies of LiH and Li_2 are calculated from the difference between the sum of the energies of the two atoms and the energy of the diatomic molecule at equilibrium. The dissociation energies obtained with the CVQZ and CV5Z basis (with no core-valence correlation) agree well with those of the VQZ and V5Z calculations in Song et al. work [4]. The core-valence correlation slightly lowers the dissociation energies with the J99 basis (by 0.25 kcal.mol^{-1} or 87 cm^{-1}), while increases them with the ACVTZ, CVQZ and CV5Z basis. The dissociation energies of LiH and Li_2 obtained with the J99 basis considering core-valence correlations agree reasonably well with the experimental data with a difference less than 1 kcal.mol^{-1} (or 350 cm^{-1}). The vertical ionization energies of LiH and Li_2 are calculated as the difference between the energies of LiH^+ and Li_2^+ at the equilibrium bond lengths of the corresponding neutral species. The ground-state energy of LiH^+ calculated with the ACVTZ, CVQZ, CV5Z and J99 basis considering the core-valence correlation are -7.7720, -7.7733 -7.7740, and -7.7693 a.u., and those of Li_2^+ are -14.7951, -14.7987, -14.8001, and -14.7911 a.u., respectively. The calculations with the J99 basis gives the closest values to the experimental ionization potentials among the four basis sets for both LiH^+ and Li_2^+. These results indicate the reliability of the present basis set and the MRCI method in predicting properties of LiH and Li_2.

Table S2: Comparison of equilibrium geometries and dissociation energies of the ground states of the LiH and Li_2 diatomic molecules

**LiH** ($X^1\Sigma^+$)	**Method**	**Basis**	**c.v.**	R_e **[a.u.]**	E_{min} **[a.u.]**	D_e **[kcal/mol]**	**I.P. [eV]**
Present	MRCI	J99	Y	3.0218	-8.0632	56.80	7.9978
	MRCI	CV5Z	Y	3.0155	-8.0694	57.70	8.0385
	MRCI	CVQZ	Y	3.0180	-8.0684	57.53	8.0293
	MRCI	ACVTZ	Y	3.0255	-8.0652	56.99	7.9999
	MRCI	J99	N	3.0388	-8.0232	57.05	7.9882
	MRCI	CV5Z	N	3.0382	-8.0245	57.55	8.0081
	MRCI	CVQZ	N	3.0389	-8.0242	57.42	8.0026
	MRCI	AVTZ	N	3.0433	-8.0232	56.87	7.9773
Yuan[a]	MRCI-F12	AVTZ	N	3.0160	-	57.99	-
Song[b]	DMBE/SEC PES		N	3.0336	-	58.11	-
	MRCI (Fitting)	V5Z	N	3.0351	-	57.57	-
	MRCI (Fitting)	VQZ	N	3.0359	-	57.43	-
Jasik [c]	MRCI	modified cc-pV5Z+ECP2 SDF	N	3.003	-	58.12	
Stwalley[d]	Exp.	-	-	3.0152	-	57.977±0.001	-
NIST	Exp.	-	-	-	-	-	7.9 ± 0.3
**Li_2** ($X^1\Sigma_g^+$)	**Method**	**Basis**	**c.v.**	R_e **[a.u.]**	E_{min} **[a.u.]**	D_e **[kcal/mol]**	**I.P. [eV]**
Present	MRCI	J99	Y	5.0594	-14.9836	23.65	5.2373
	MRCI	CV5Z	Y	5.0543	-14.9931	24.06	5.2524
	MRCI	CVQZ	Y	5.0571	-14.9916	24.01	5.2496
	MRCI	ACVTZ	Y	5.0652	-14.9872	23.87	5.2274
	MRCI	J99	N	5.0954	-14.9030	23.74	5.2017
	MRCI	CV5Z	N	5.0994	-14.9038	24.07	5.2124
	MRCI	CVQZ	N	5.0994	-14.9037	24.04	5.2112
	MRCI	AVTZ	N	5.1035	-14.9033	23.81	5.2035
Yuan[a]	MRCI-F12	AVTZ	N	5.0531	-	28.58	-
Song[b]	DMBE/SEC PES	-	N	5.0877	-	24.45	-
	MRCI(Fitting)	V5Z	N	5.1020	-	24.10	-
	MRCI(Fitting)	VQZ	N	5.1046	-	24.04	-
Jasik [e]	MRCI	modified cc-pV5Z+ECP	N	5.0229	-	24.63	-
Verma	Experiment	-	-	5.0586[f]	-	24.35±0.01[g]	-
McGeogh[h]	Experiment	-	-	-	-	-	5.1127 ± 0.0003

[a] Ref. [7] , [b] Ref. [4], [c] Ref. [5], [d] Ref. [8], [e] Ref. [6], [f] Ref. [9], [g] Ref. [10], [h] Ref. [11]

4. Fortran routines of the MSA PES

4.1. Global PES

- fortran_files/Global_PES/pes_Li2H.f90 : modules to calculate the Li_2H potential energy
- fortran_files/Global_PES/coeff.dat : coefficients data file read by pes_Li2H.f90
- fortran_files/Global_PES/driver_Li2H.f90 : example main program to run pes_Li2H.f90
- fortran_files/Global_PES/example.inp : example input file
- fortran_files/Global_PES/example.out : example output file

4.2. Local PES

Same file names but in the fortran_files/Local_PES/ directory.

References

1. Yiannopoulou, A.; Jeung, G.-H.; Park, S.J.; Lee, H.S.; Lee, Y.S. Undulations of the potential-energy curves for highly excited electronic states in diatomic molecules related to the atomic orbital undulations. *Phys. Rev. A* **1999**, *59*, 1178–1186.
2. Kelly, R.L. Atomic and ionic spectrum lines below 2000 angstroms; hydrogen through krypton part I (H-Cr). *J. Phys. Chem. Ref. Data* **1987**, *16*, 1–649.
3. Prascher, B.P.; Woon, D.E.; Peterson, K.A.; Dunning, T.H.; Wilson, A.K. Gaussian basis sets for use in correlated molecular calculations. VII. Valence, core-valence, and scalar relativistic basis sets for Li, Be, Na, and Mg. *Theor. Chem. Acc.* **2011**, *128*, 69–82.
4. Song, Y.-Z.; Li, Y.-Q.; Gao, S.-B.; Meng, Q.-T. Accurate ab initio-based DMBE potential energy surface for $HLi_2(X^2A')$ via scaling of the external correlation. *Eur. Phys. J. D* **2014**, *68*.
5. Jasik, P.; Sienkiewicz, J.E.; Domsta, J.; Henriksen, N.E. Electronic structure and time-dependent description of rotational predissociation of LiH. *Phys. Chem. Chem. Phys.* **2017**, *19*, 19777–19783.
6. Jasik, P.; Sienkiewicz, J.E. Calculation of adiabatic potentials of Li_2. *Chem Phys* **2006**, *323*, 563.
7. Yuan Meiling; Li Wentao; Chen Maodu Global X^2A' potential energy surface of Li_2H and quantum dynamics of $H + Li_2\ (X^1\Sigma_g^+) \rightarrow Li + LiH\ (X^1\Sigma^+)$ reaction. *International Journal of Quantum Chemistry* **2017**, *117*, e25380.
8. Stwalley, W.C.; Zemke, W.T. Spectroscopy and Structure of the Lithium Hydride Diatomic Molecules and Ions. *J. Phys. Chem. Ref. Data* **1993**, *22*, 87–112.
9. Verma, K.K.; Koch, M.E.; Stwalley, W.C. New Observations and Mass-Reduced Analyses of the Laser-Excited Fluorescence of the $B^1\Pi_u$-$X^1\Sigma_g^+$ Bands of the $^6Li^7Li$ Molecule. *J. Mol. Spectrosc.* **1981**, *87*, 548–559.
10. Verma, K.K.; Koch, M.E.; Stwalley, W.C. Observation of Levels near Dissociation in the $X^1\Sigma_g^+$ State of $^7Li_2$. *J. Chem. Phys.* **1983**, *78*, 3614–3622.
11. McGeoch, M.W.; Schlier, R.E. Autoionizing Rydberg States of. The Li_2 Molecule: Molecular Constants for Li_2^+. *Chem. Phys. Lett.* **1983**, *99*, 347–352.

© 2018 by the authors. Submitted for possible open access publication under the terms and conditions of the Creative Commons Attribution (CC BY) license (http://creativecommons.org/licenses/by/4.0/).
